# Prenatal Fine Particulate Matter, Maternal Micronutrient Antioxidant Intake, and Early Childhood Repeated Wheeze: Effect Modification by Race/Ethnicity and Sex

**DOI:** 10.3390/antiox11020366

**Published:** 2022-02-11

**Authors:** Yueh-Hsiu Mathilda Chiu, Kecia N. Carroll, Brent A. Coull, Srimathi Kannan, Ander Wilson, Rosalind J. Wright

**Affiliations:** 1 Department of Environmental Medicine and Public Health, Icahn School of Medicine at Mount Sinai, One Gustave L. Levy Place, P.O. Box 1057, New York, NY 10029, USA; mathilda.chiu@mssm.edu (Y.-H.M.C.); kecia.carroll@mssm.edu (K.N.C.); 2 Kravis Children’s Hospital, Department of Pediatrics, Icahn School of Medicine at Mount Sinai, New York, NY 10029, USA; 3 The Institute for Exposomic Research, Icahn School of Medicine at Mount Sinai, New York, NY 10029, USA; 4 Department of Biostatistics, Harvard TH Chan School of Public Health, Harvard University, Boston, MA 02115, USA; bcoull@hsph.harvard.edu; 5 Division of Metabolism, Endocrinology and Diabetes, Department of Internal Medicine, University of Michigan, Ann Arbor, MI 48105, USA; srimathik@gmail.com; 6 Department of Statistics, Colorado State University, Fort Collins, CO 80523, USA; ander.wilson@colostate.edu

**Keywords:** prenatal air pollution exposure, childhood wheeze, antioxidant intake, race and ethnicity, sex difference, developmental origins of health and disease

## Abstract

Fine particulate matter (PM_2.5_) potentiates in utero oxidative stress influencing fetal development while antioxidants have potential protective effects. We examined associations among prenatal PM_2.5_, maternal antioxidant intake, and childhood wheeze in an urban pregnancy cohort (*n* = 530). Daily PM_2.5_ exposure over gestation was estimated using a satellite-based spatiotemporally resolved model. Mothers completed the modified Block98 food frequency questionnaire. Average energy-adjusted percentile intake of β-carotene, vitamins (A, C, E), and trace minerals (zinc, magnesium, selenium) constituted an antioxidant index (AI). Maternal-reported child wheeze was ascertained up to 4.1 ± 2.8 years. Bayesian distributed lag interaction models (BDLIMs) were used to examine time-varying associations between prenatal PM_2.5_ and repeated wheeze (≥2 episodes) and effect modification by AI, race/ethnicity, and child sex. Covariates included maternal age, education, asthma, and temperature. Women were 39% Black and 33% Hispanic, 36% with ≤high school education; 21% of children had repeated wheeze. Higher AI was associated with decreased wheeze in Blacks (OR = 0.37 (0.19–0.73), per IQR increase). BDLIMs identified a sensitive window for PM_2.5_ effects on wheeze among boys born to Black mothers with low AI (at 33–40 weeks gestation; OR = 1.74 (1.19–2.54), per µg/m^3^ increase in PM_2.5_). Relationships among prenatal PM_2.5_, antioxidant intake, and child wheeze were modified by race/ethnicity and sex.

## 1. Introduction

Early life wheezing respiratory illnesses account for significant morbidity and health care utilization [1]. Episodic wheezing frequently precedes asthma and is related to reduced lung function with potential lifelong consequences [2]. A key step in identifying children at risk for chronic respiratory disorders is characterizing risk factors and mechanisms that lead to early predisposition. The high prevalence and substantial costs of these disorders have also motivated efforts to identify factors that mitigate risk. Wheezing respiratory illnesses and asthma have their roots in utero [3]; thus, identifying modifiable or mitigating factors in critical windows of development can inform prevention strategies.

Factors gaining attention with respect to fetal programming of wheezing in infancy and early childhood include particulate air pollution [4,5,6,7,8] and nutritional factors [9,10,11]. Programming is a consequence of environmental and nutritional exposures during critical life periods affecting the physiological system that orchestrates underlying developmental processes. While research has largely been focused on how ambient air pollution and diet independently affect children’s health, evolving theory underscores the importance of studying their interactive effects [12]. Mechanisms responsible for adverse respiratory outcomes associated with particulate pollution are not fully elucidated; however, oxidative stress is thought to play a central role [13,14]. Diet is a major source of antioxidants, which may alleviate the effects of PM_2.5_ exposure during pregnancy [15]. Maternal dietary factors that reduce fetal vulnerability to oxidative stress may modify air pollution effects on early childhood wheeze programming, although this has not been studied. Limited studies that attempted to examine the interactions between prenatal PM_2.5_ and antioxidant intake on respiratory health were conducted in animal/in vitro models [16] or in adolescents [17]. Furthermore, previous studies examining the effect of antioxidant intake on respiratory health at other life stages have mostly considered each antioxidant micronutrient one at a time separately in their analyses, while very few considered an overall or composite nutrient index [17].

Additional parameters may further modify these relationships. Racial and ethnic minority children living in economically disadvantaged communities are particularly burdened by wheeze and asthma, in part due to co-occurring environmental exposures and socioeconomic influences [18,19]. Prior work has also consistently revealed sex-specific effects of prenatal pollution exposure on child respiratory outcomes [20,21], as well as sexually dimorphic effects of prenatal antioxidants on child health [22]. However, limited studies have considered higher-order effect modification by both race/ethnicity and child sex. Studies that examine these more complex interactions on early childhood wheezing respiratory illness may enhance our ability to identify those at greatest risk.

To begin to address these complexities, we leveraged an ethnically diverse lower socioeconomic status (SES) urban pregnancy cohort to examine associations among prenatal PM_2.5_ exposure, maternal antioxidant intake, and childhood repeated wheeze. Specifically, we first examined whether maternal overall antioxidant intake as indexed by a composite level modified or attenuated the effect of prenatal PM_2.5_ on early childhood repeated wheeze. Next, in order to identify potential susceptible subgroups, we examined whether these associations were further modified by race/ethnicity and fetal sex. These complex associations were examined using data-driven approaches to identify potential sensitive exposure windows.

## 2. Materials and Methods

Participants were from the PRogramming of Intergenerational Stress Mechanisms (PRISM) longitudinal pregnancy cohort designed to examine associations between prenatal stress, nutrition, and other environmental factors and child developmental outcomes. This dual-site study enrolled *n* = 923 women receiving prenatal care from the Beth Israel Deaconess Medical Center and East Boston Neighborhood Health Center in Boston, MA, USA (March 2011–December 2013), and Mount Sinai Hospital in New York City, NY, USA (April 2013–August 2019). Eligibility criteria included English- or Spanish-speaking, ≥18 years of age, and singleton pregnancy. Exclusions included maternal intake of ≥7 alcoholic drinks/week prior to pregnancy recognition or any after pregnancy recognition, HIV positive status, and congenital abnormalities that could impact ongoing participation. The analytic sample for this study includes *n* = 530 mother–child dyads enrolled at 22.3 ± 9.1 weeks gestation with complete data on prenatal daily ambient PM_2.5_ exposure, maternal dietary intake, and children’s repeated wheeze. Those enrolled were similar to those in the analytic sample based on maternal age at delivery, education, and asthma history and child sex (*p*-values > 0.1); there were more Black/Hispanic Black (40.6% vs. 38.7%) and non-Black Hispanic (34.6% vs. 33.0%) among those enrolled versus the analytic sample (*p* = 0.03, Online Supplement, Table S1). Study procedures were reviewed and approved by the relevant institutions’ human studies committees; mothers provided written consent in their primary language.

### 2.1. Maternal Antioxidant Intake

At approximately mid-pregnancy, mothers reported their usual dietary and supplement intake over the prior three months by completing the modified Block98 Food Frequency Questionnaire (FFQ), administered in English or Spanish. The list of the food items, originally based on the National Health and Nutrition Examination Survey III dietary recall [23], was modified to include a more comprehensive list of fish and seafood [24]. For each food/beverage item, mothers reported how frequent (rarely/never, daily, weekly, monthly) and how much (small, medium, or large serving with portion size pictures provided) they generally consumed. Type and frequency of vitamins, minerals, and other dietary supplements taken during pregnancy were also reported. FFQ data were processed using the online Block Dietary Data Systems (Berkeley, CA, USA). To derive a composite index of antioxidant intake, we considered seven micronutrients, namely β-carotene; magnesium; selenium; zinc; and vitamins A, C, and E. These seven micronutrients are well-established antioxidant compounds that meet the evidence-based criteria set forth by the Institute of Medicine for dietary antioxidants [25,26] (see Online Supplement for greater detail). Further, our group has previously validated FFQ estimates of antioxidant intake for these micronutrients, demonstrating reasonable correlations between FFQ reports and 24 h dietary recalls in PRISM participants [27]. To standardize nutrients by adjusting for total daily energy intake (kcal), we used the residual method as done previously [28]. We then converted the distribution of each energy-adjusted micronutrient intake into a percentile based on the relative ranks across participants. The percentiles of the seven micronutrients were then averaged to derive an antioxidant index (AI) for each participant [29]. Higher AI scores reflect greater antioxidant intake across the seven micronutrients.

### 2.2. Prenatal PM_2.5_ Exposure

Exposure levels of ambient PM_2.5_ were estimated using a hybrid satellite-based spatiotemporal prediction model, as detailed previously [30]. Briefly, daily surface PM_2.5_ measurements (obtained by U.S. Environmental Protection Agency Air Quality System and Interagency Monitoring of Protected Visual Environments Network) were regressed on satellite-derived aerosol optical depth (AOD) measurements (1 km^2^ spatial resolution). To determine residence-specific daily PM_2.5_, meteorological variables and land-use terms in machine learning algorithms were included to minimize the prediction error and estimate the daily exposures. Daily prediction models were calibrated, and estimates were validated with a robust out of sample 10-fold cross-validation (R^2^ = 0.87). Each participant’s prenatal daily exposure to PM_2.5_ was derived based on the calendar dates and residential address across pregnancy and updated if they moved. Daily exposure levels were aggregated into weekly averaged levels to reduce noise in the daily estimates. For those born prior to 40 weeks gestation, exposure for the remaining weeks was based on postnatal PM_2.5_ estimates corresponding to this time.

### 2.3. Repeated Wheeze

Maternal-reported child wheeze was ascertained at approximately 4-month intervals from birth to age 3 years, and then annually thereafter, through telephone and face-to-face follow-up visits. Mothers were asked, “Since we last spoke with you on (date), has your baby had wheezing or whistling in the chest?” or “Has your child ever had wheezing or whistling in the chest in the past 12 months?”, as appropriate. Repeated wheeze was defined as child wheeze reported two or more times. The average follow-up period of the study participants was 4.1 ± 2.8 years.

### 2.4. Covariates

Women reported age, race/ethnicity, and education at enrollment; child’s sex, birthweight, and date of birth were extracted from medical records. Gestational age at birth was extracted from medical records; if not available, it was then derived based on (1) difference between date of delivery and self-reported last menstrual period and (2) ultrasound estimates from the first-trimester examination. Sex-specific birthweight for gestational age z-scores were calculated based on the Fenton growth charts [31]. Maternal asthma history was based on report of ever having asthma and/or report of medication or healthcare utilization for asthma. Information on prenatal maternal smoking and secondhand smoke exposure was also reported by mothers prenatally. In the analysis, race/ethnicity was categorized as “Black” (Black and Hispanic Black), “Hispanic” (non-Black Hispanic), “White” (non-Hispanic White), and “Other”. Prenatal daily temperature was derived as previously described [32].

### 2.5. Statistical Analysis

The frequencies and distributions of covariates for the sample as a whole and based on maternal antioxidant intake were derived. We examined the main effects of maternal antioxidant intake and prenatal PM_2.5_ exposure on children’s repeated wheeze in separate models. We used multivariable-adjusted logistic regressions to examine the main effects of maternal AI score, considered as a continuous variable as well as a binary variable (high vs. low intake based on median split), on repeated wheeze in the overall sample as well as stratified by maternal race/ethnicity. Given that averaging PM_2.5_ exposure across gestation or sampling exposure at an arbitrary time point may result in biased estimates or missing existing associations [33], we estimated the time-varying associations between prenatal weekly averaged PM_2.5_ and repeated wheeze using a distributed lag model framework, a data-driven approach that takes into account the effects and correlations among exposures at different time points by creating an exposure lag space using participant’s weekly exposure estimates throughout gestation. We estimated antioxidant-specific (high vs. low intake) and sex-specific time-varying associations between weekly PM_2.5_ exposure and repeated wheeze using Bayesian distributed lag interaction models (BDLIMs) [34]. This approach assumes that PM_2.5_ effects in any given exposure timepoint are linear but allows effects to vary nonlinearly across exposure timepoints. We first fit models assuming a common distributed lag effect for all subjects, and then fit distributed lag interaction models to examine differences in both the magnitude and timing of effects by maternal AI (high vs. low), as well as by child sex (see Online Supplement for greater detail). In secondary analyses, we conducted multivariable-adjusted logistic regression models examining the associations between PM_2.5_ levels averaged over the critical windows identified by the BDLIMs and repeated wheeze. Stratified analyses were conducted to further examine effect modification by maternal race/ethnicity for all models. Covariates included maternal race/ethnicity, age, education, and asthma history, as well as child sex and prenatal averaged temperature. Further, sensitivity analyses were conducted to additionally adjust for prenatal maternal smoking, secondhand smoke exposure, postnatal PM_2.5_ exposure averaged over the first year of the child’s life, and study site (Boston vs. New York), as well as a potential pathway variable, birthweight for gestational age z-score. Analyses were conducted using the “*regimes*” package in *R* (v4.0.3, Vienna, Austria), as well as SAS statistical software (v9.4, SAS Institute Inc., Cary, NC, USA).

## 3. Results

**Figure 1:**
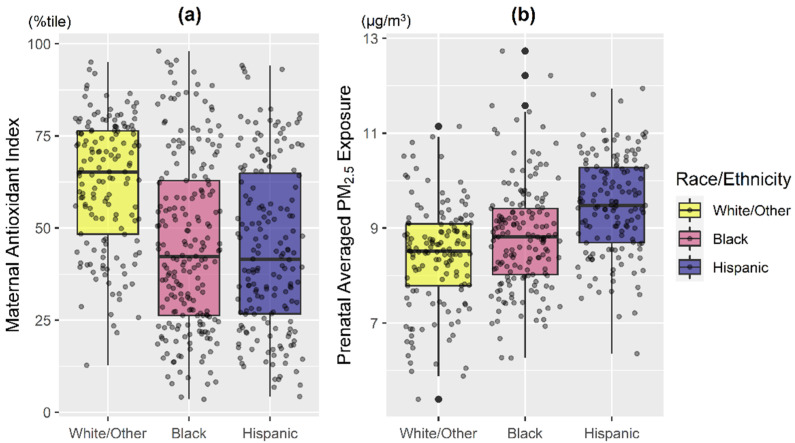
**Distribution of maternal antioxidant intake and prenatal PM_2.5_ exposure averaged across pregnancy by maternal race/ethnicity.** Boxplots of (**a**) maternal composite antioxidant index and (**b**) prenatal PM_2.5_ exposure averaged across pregnancy, stratified by maternal race/ethnicity (Black: Black/Hispanic Black; Hispanic: non-Blank Hispanic; White/Other: non-Hispanic White/other race). Bottom line and upper line of the box denote 25th percentile (Q1) and 75th percentile (Q3) of the distribution, while the thick line within the box denotes median. Lower and upper whiskers of the vertical line denote Q1 − 1.5*IQR and Q3 + 1.5*IQR, while the dark black dots represent potential extreme values. IQR: interquartile range. Kruskal–Wallis test indicated that maternal antioxidant index and averaged prenatal PM_2.5_ among racial/ethnic subgroups are significantly different (both *p* < 0.001).

The majority of women were ethnic minorities (38.7% Black, 33.0% Hispanic) and 35.5% had ≤12 years of education (Table 1). Mothers with lower antioxidant intake were younger and reported less education (Table 1; both *p* < 0.01). Overall, mothers in the White/Other group had generally higher antioxidant intake and lower PM_2.5_ exposure compared to those identifying as Black and Hispanic (Figure 1; *p* < 0.001). Hispanic women had the highest averaged PM_2.5_ exposure level, followed by Black (Figure 1; *p* < 0.001).

### 3.1. Main Effects of Prenatal Antioxidant Intake and PM_2.5_ on Repeated Wheeze

**Figure 2:**
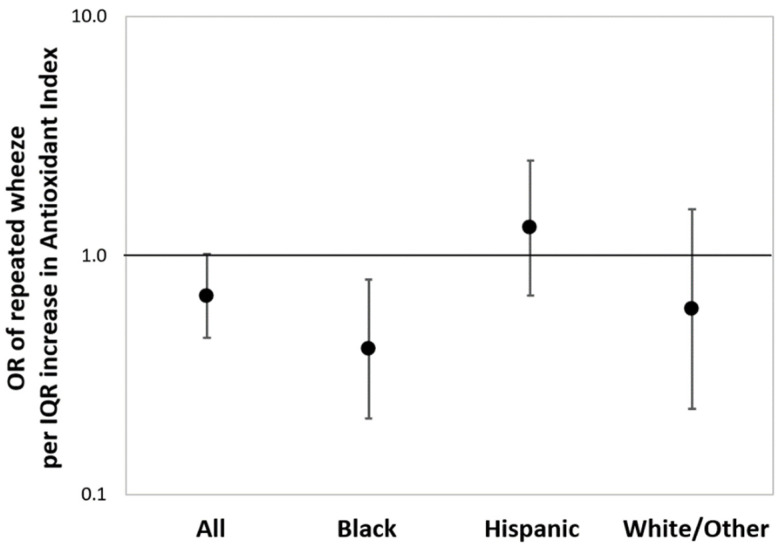
**Associations between maternal antioxidant index (AI) score and children’s repeated wheeze.** Results from multivariable-adjusted logistic regressions examining associations between maternal AI score and children’s repeated wheeze in the sample overall and stratified by race/ethnicity (Black: Black/Hispanic Black; Hispanic: non-Blank Hispanic; White/Other: non-Hispanic White/other race). Solid dot denotes odds ratio (OR) of repeated wheeze per interquartile range (IQR) increase in AI score, and the error bars denote 95% confidence interval (95% CI). The models were adjusted for child sex, maternal age at delivery, education status, maternal history of asthma (and race/ethnicity in the overall model). IQR of antioxidant index: 39.3 (percentile).

Figure 2 shows the results from multivariable-adjusted logistic regressions estimating the associations between maternal AI score as a continuous variable and children’s repeated wheeze, adjusting for child sex, maternal age, education, and asthma history (and race/ethnicity in the overall model). In the sample overall, a higher maternal AI score was associated with reduced repeated wheeze, although significance was borderline (OR = 0.68, 95% CI: 0.45–1.03; per IQR increase in AI score). Increased maternal AI score was most significantly associated with decreased repeated wheeze among children in the Black group (OR = 0.37, 95% CI: 0.19–0.73; per IQR increase in AI score). When treating antioxidant intake as categorical (high vs. low), association patterns were similar to the models using continuous AI, although the statistical significance was attenuated (Online Supplement, Figure S1). BDLIMs did not identify a significant exposure window between prenatal PM_2.5_ and repeated wheeze in the main effects model considering the overall sample (Online Supplement, Figure S2).

### 3.2. Prenatal PM_2.5_ and Repeated Wheeze: Effect Modification by AI, Race/Ethnicity, and Child Sex

**Figure 3:**
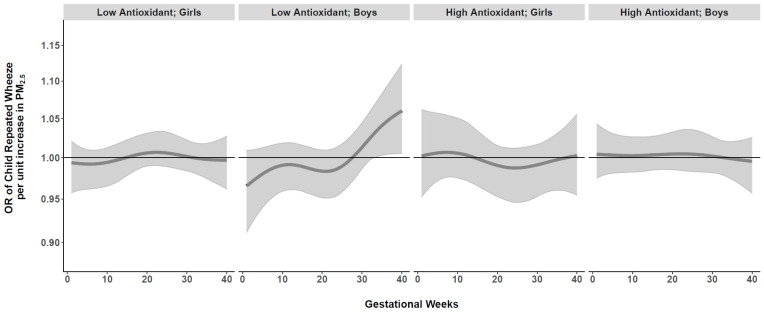
**Antioxidant- and sex-specific time-varying odds ratios (95% CIs) of child’s repeated wheeze per µg/m^3^ increase in prenatal weekly averaged PM_2.5_ levels across gestation in Black mothers.** Antioxidant- and sex-specific time-varying associations between prenatal weekly PM_2.5_ exposure and children’s repeated wheeze were estimated by a BDLIM among Black mothers, adjusting for maternal age at delivery, education, asthma history, and prenatal averaged temperature. The x-axis demarcates gestational age in weeks. The y-axis represents the odds ratio (OR) of repeated wheeze per 1 µg/m^3^ increase in prenatal PM_2.5_ exposure. The solid line represents the predicted effect estimate, and the gray area indicates the 95% confidence interval (CI). A significant exposure window is identified for the time periods where the estimated pointwise 95% CI (shaded area) does not include 1. Results indicated a significant exposure window at 33–40 weeks gestation in boys born to Black mothers with low antioxidant intake.

We next conducted BDLIMs to examine interactions among time-varying prenatal PM_2.5_ exposure, maternal antioxidant intake (high vs. low), and child sex. When examining effect modification by maternal AI, there was a suggested association between PM_2.5_ exposure and repeated wheeze for the low antioxidant group in Blacks, but no statistically significant exposure window was identified (Online Supplement, Figure S3). We did not observe significant effect modification by child sex in the overall sample (Online Supplement, Figure S4). However, when we examined joint interactions by both maternal AI and child sex together, BDLIMs identified a significant exposure window at 33–40 weeks gestation in boys born to Black mothers with low antioxidant intake (Figure 3). Based on the posterior model probability and DIC, the BDLIM indicated that both the magnitude and the sensitive windows of the time-varying association between prenatal PM_2.5_ and repeated wheeze were different for each group of maternal antioxidant–child sex combinations in the Black group. We did not find significant exposure windows in Hispanics or Whites (Online Supplement, Figure S5).

**Figure 4:**
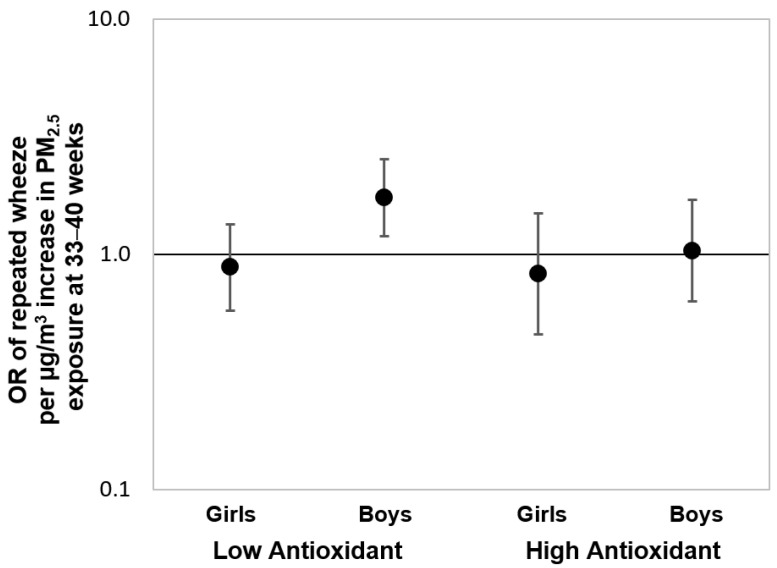
**Odds ratios (95% CIs) of child’s repeated wheeze corresponding to PM_2.5_ exposure at 33–40 weeks gestation in Black mothers, stratified by antioxidant intake and child sex.** Results from multivariable-adjusted logistic regressions examining the associations between prenatal PM_2.5_ exposure level averaged over 33–40 weeks gestation (i.e., the sensitive exposure window identified by BDLIM) and offspring’s repeated wheeze among Black mothers, stratified by antioxidant intake status (high vs. low intake categorized by median split) and child sex. The models were adjusted for maternal age at delivery, maternal education, maternal history of asthma, and prenatal averaged temperature.

To further assess the associations over the “sensitive windows” observed in the Black group, we also fit multivariable-adjusted logistic regression models using PM_2.5_ averaged over gestational weeks 33–40 as identified by the BDLIM. Figure 4 shows the results of these logistic regressions stratified by maternal antioxidant intake and child sex. We observed a significant effect estimate between increased PM_2.5_ exposure during the identified sensitive window and elevated risk of repeated wheeze among boys born to Black women with lower prenatal antioxidant intake (OR = 1.74, 95% CI: 1.19–2.54; per µg/m^3^ increase in PM_2.5_). Notably, no significant associations were observed when logistic regression models were fit using PM_2.5_ averaged over the entire gestational period (Online Supplement, Figure S6), highlighting the importance of identifying the most relevant exposure window otherwise associations may be missed.

Finally, we conducted sensitivity analyses by additionally adjusting for (1) prenatal maternal smoking, secondhand smoke exposure, and averaged postnatal PM_2.5_ level over the first year of the child’s life (Online Supplement, Figure S7) and (2) study site, birthweight for gestational age z-score, and season of birth (Online Supplement, Figure S8). These models were materially unchanged.

## 4. Discussion

In this multiethnic inner-city population, we found that increased prenatal PM_2.5_ exposure during late pregnancy was associated with early childhood repeated wheeze, particularly in boys born to Black mothers with low antioxidant intake. While this supports our *a priori* hypothesis that greater intake of antioxidants in pregnant women attenuates effects of PM_2.5_ on early childhood repeated wheeze, associations were only evident when accounting for effect modification by additional characteristics including race/ethnicity and child sex.

Our findings may help explain conflicting reports in the literature examining associations between in utero air pollution exposure and early wheeze. We previously linked higher exposure of PM_2.5_ averaged over pregnancy with increased repeated wheeze by age 2 years in a pregnancy cohort in the Northeastern United States (US) [4]. A study in Mexico considered PM_2.5_ exposure averaged over each trimester and did not find a main effect of PM_2.5_ on ever wheeze or wheeze in the past 12 months in 4-year-olds [35]. In a prospective pregnancy cohort in Kraków, Poland, using maternal PM_2.5_ exposure measured during the second trimester, a significant association with wheezing duration (number of days) in the first 2 years of life was observed [5], but it did not hold up when children were reassessed at age 3–4 years [36]. We acknowledge that potential reasons for mixed results include different outcome definitions (e.g., ever wheeze, current wheeze, repeated wheeze, days of wheezing), varied follow-up periods, and different approaches to measuring or modeling exposure. Notably, we took advantage of highly temporally resolved prenatal PM_2.5_ exposure and used advanced data-driven statistical methods that provide enhanced power to detect associations [33], and we did not find a significant association between prenatal PM_2.5_ and repeated wheeze in the main effects model. Significant findings emerged when accounting for higher-order interactions among maternal antioxidant intake, race/ethnicity, and child sex. Thus, conflicting results in prior studies examining associations between PM_2.5_ and child wheeze may also be due to a lack of consideration of important modifying factors.

To our knowledge, no prior population-based study has examined the potential protective effects of prenatal maternal antioxidant intake against effects of PM_2.5_ exposure on early childhood repeated wheeze. Overlapping evidence supports a protective role of antioxidants on respiratory effects of ambient pollutants [37]. Human studies considering respiratory health outcomes have been largely focused on adults, suggesting that vitamins C and E may help protect against pollution damage that can trigger chronic respiratory illnesses [38]. Epidemiological studies report that increased maternal antioxidant intake in pregnancy may protect against effects of ambient PM_2.5_ on other developmental outcomes and risk biomarkers, such as newborn leukocyte telomere length [39], infant neurobehavior [40], and allergic disease in adolescents [41]. Recent animal studies show that the antioxidant vitamins C and E protect against lung inflammation due to PM_2.5_ exposure in mice [42], but studies considering prenatal exposure remain limited.

Studies examining sex differences in associations between prenatal air pollution exposure and children’s health including respiratory outcomes generally suggest that boys might be more vulnerable [13,20,21,43]. The etiology of sex-specific air pollutant effects on programming of respiratory disorders remains unclear, although these data add support to the role of differential vulnerability to oxidative stress [44]. Research shows sex-specific patterns in particulate-induced oxidative stress that may disrupt molecular processes involved in lung maturation including telomere length, mitochondrial aging, epigenetics, and gene expression [45,46,47,48]. Sex differences in antioxidant defense, metabolizing enzymes, and placental responses also may play a role [49]. Effect modification by race/ethnicity may reflect increased oxidative stress documented among minoritized groups, particularly Blacks [50]. Future work is needed to elucidate mechanisms underlying the observed associations.

This study has notable strengths. Our urban multiethnic population provides an opportunity to more comprehensively examine the role of race/ethnicity. We implemented advanced statistical approaches to identify sensitive windows of prenatal air pollution exposure while considering multiway effect modifications [33]. The identification of race- and sex-specific windows of vulnerability may help elucidate the etiology of racial and sex differences in the expression of wheeze or other respiratory outcomes, which remain poorly understood [51,52]. Moreover, previous studies linking prenatal maternal intake of vitamin C [53,54], vitamin E [53,55], zinc [55], and selenium [56,57] to reduced risk of wheezing mostly examined each micronutrient in separate models, while some studies found significant associations with some antioxidants but not all (e.g., beneficial effects observed for vitamin C but not vitamin E or zinc [54]). Given prior documentation of racial/ethnic differences in dietary intake [27], we considered a composite index of seven antioxidant micronutrients in this multiethnic sample and demonstrated an inverse association between the maternal antioxidant index and repeated wheeze.

We also acknowledge some limitations. We did not have data on indoor or personal particulate matter exposures, and thus there may be exposure measurement error, which could potentially affect precision and induce bias in effect estimates [58]. One of the main goals of this study was to identify sensitive exposure windows across pregnancy that are most relevant to wheezing programming, which requires highly temporally resolved PM_2.5_ exposure data; however, it is costly implausible and operationally infeasible to collect daily or weekly personal sampling data throughout the entire pregnancy period from all the participants. Our use of comprehensive, temporally and spatially resolved models to estimate residential particulate pollutant exposures for each participant is the optimal approach in this epidemiological study design because it likely minimizes misclassification relative to personal sampling studies that collected data only at convenient timepoints during pregnancy. Although the exposure prediction model did not account for indoor or personal sources as well as the indoor–outdoor penetration rate of the particulate matter, previous studies have demonstrated a reasonable contribution of outdoor air pollution to indoor pollutant levels and monitored personal exposures [59,60,61]. Moreover, a major source of indoor particulate matter is tobacco smoke exposure, and our sensitivity analyses further adjusting for maternal and secondhand smoke exposure did not substantively change our results. While we adjusted for a number of important covariates in primary and sensitivity analyses, we cannot rule out the possibility of confounding by other unmeasured factors. While we found significant results only in the Black group, it is possible that we did not have an adequate sample size to detect all associations. Suggested sex- and race-specific effects warrant future follow-up in larger cohorts. Although child’s repeated wheeze status is a commonly used measurement for early childhood respiratory symptoms, it was self-reported by mothers, and thus we cannot rule out potential misclassification, which is likely nondifferential. Notably, questionnaire report of wheeze has been validated through a number of studies correlating the relationship of persistent wheeze to subsequent diagnosis of asthma, lung function, and airway hyperresponsiveness [62,63]. While micronutrient intake was estimated using maternal self-report on FFQs which may be vulnerable to recall bias, we note prior literature demonstrating acceptable validity and reproducibility which are stable across pregnancy when using this approach to estimate usual dietary intake [64,65,66]. In addition, maternal FFQ antioxidant intake was validated using 24 h dietary recalls in the PRISM sample used in these analyses [27].

## 5. Conclusions

In summary, this study found that increased exposure to prenatal PM_2.5_ may have sex-specific and time-dependent effects that varied by race/ethnicity and maternal antioxidant intake. Air pollution remains a major pediatric public health focus because of its ubiquity and projected increase in exposure patterns over the coming years due to climate change [67,68], and it remains an urgent public health challenge contributing to the growing prevalence of childhood respiratory disorders [7,69]. Targeting oxidative stress with antioxidant supplements has attracted attention as a strategy to mitigate the harmful effects of air pollution. In many urban cities in the US and emerging economies where air pollution is worsening and difficult to avoid, this approach may be the most feasible option to protect individuals.

## Figures and Tables

**Table 1:** Participant characteristics: PRISM study.

	Analytic Sample Overall		Antioxidant Intake ^b^			
	(*n* = 530)		Low (*n* = 269)		High (*n* = 271)	
**Child sex (*n*, %)**						
Girls	254	47.9	133	50.2	121	45.7
Boys	276	52.1	132	49.8	144	54.3
**Child repeated wheeze (*n*, %)**						
No	420	79.3	205	77.4	215	81.1
Yes	110	20.8	60	22.6	50	18.9
**Maternal age at delivery**						
Age in years (median, IQR **^a^**)	30.2	(25.3–34.2)	27.6	(23.7–32)	32.2	(28.6–35.5)
**Maternal education (*n*, %)**						
>12 years (more than high school)	342	64.5	143	54.0	199	75.1
≤12 years (high school or less)	188	35.5	122	46.0	66	24.9
**Maternal race/ethnicity (*n*, %)**						
Black (Black/Hispanic Black)	205	38.7	121	45.7	84	31.7
Hispanic (non-Black Hispanic)	175	33.0	105	39.6	70	26.4
White (non-Hispanic White)	120	22.6	26	9.8	94	35.5
Other ^c^	30	5.7	13	4.9	17	6.4
**Mother asthma (*n*, %)**						
No	372	70.2	183	69.1	189	71.3
Yes	158	29.8	82	30.9	76	28.7
**Maternal antioxidant intake**						
Antioxidant index (AI; median, IQR **^a^**)	49.5	(31.3–70.6)	31.3	(21.9–39.9)	70.6	(58.7–77.9)
**Prenatal daily PM_2.5_ exposure**						
Prenatal average (µg/m^3^; median, IQR **^a^**)	8.9	(8.2–9.6)	9.1	(8.3–9.9)	8.7	(8–9.4)
**Prenatal daily temperature**						
Prenatal average (°C; median, IQR **^a^**)	12.1	(9.6–14.5)	12.4	(9.9–14.7)	11.8	(9.3–14.4)

**^a^** IQR = interquartile range (25th percentile–75th percentile). **^b^** Low vs. high antioxidant intake status was categorized by median split of maternal antioxidant index (AI) score. Mothers with lower antioxidant intake were on average younger, with less education, and more likely to be Black or Hispanic (all *p* < 0.01); other characteristics including maternal asthma history, child sex, prenatal PM_2.5_, and temperature levels are similar between the two antioxidant intake groups. ^c^ Mothers identified themselves as Asian (*n* = 14), Native Hawaiian or other Pacific Islander (*n* = 1), American Indian/Alaska Native (*n* = 1), multiple races (*n* = 12), or other (*n* = 2).

## Data Availability

The data presented in this study are available on request from the corresponding author. The data are not publicly available due to their containing confidential and protected health information that could compromise the privacy of research participants.

## Supplementary Materials

The following supporting information can be downloaded at: https://www.mdpi.com/article/10.3390/antiox11020366/s1. Detailed descriptions on the selection of seven antioxidant micronutrients that are used to derive the composite antioxidant index score; Detailed descriptions on the Implementation of BDLIMs; Table S1: Comparison between participants enrolled in the PRISM study and those included in the current analysis; Figure S1: Associations between maternal antioxidant intake status (high vs. low) and children’s repeated wheeze; Figure S2: Time-varying odds ratio (95% CI) of repeated wheeze corresponding to per µg/m^3^ increase in prenatal weekly PM_2.5_ levels; Figure S3: Antioxidant-specific time-varying odds ratios (95% CIs) of repeated wheeze corresponding to per µg/m^3^ increase in prenatal weekly PM_2.5_ levels among children born to Black mothers; Figure S4: Sex-specific time-varying odds ratios (95% CIs) of repeated wheeze corresponding to per µg/m^3^ increase in prenatal weekly PM_2.5_ levels; Figure S5: Antioxidant- and sex-specific time-varying odds ratios (95% CIs) of repeated wheeze corresponding to per µg/m^3^ increase in prenatal weekly PM_2.5_ levels among children born to Hispanic mothers and White mothers, as compared to those born to Black mothers; Figure S6: Odds ratios (95% CIs) of offspring’s repeated wheeze corresponding to PM_2.5_ exposure averaged over the entire gestational period in Black mothers, stratified by antioxidant intake and child sex; Figures S7 and S8: Antioxidant- and sex-specific time-varying odds ratios (95% CIs) of repeated wheeze corresponding to per µg/m^3^ increase in prenatal weekly PM_2.5_ levels among children born to Black mothers—sensitivity analyses adjusting for additional covariates.
