# Supplementary material for: Prenatal Fine Particulate Matter, Maternal Micronutrient Antioxidant Intake, and Early Childhood Repeated Wheeze: Effect Modification by Race/Ethnicity and Sex
Source: Antioxidants (Basel). 2022 Feb 11;11(2):366. doi: 10.3390/antiox11020366 (PMC8868511; doi:10.3390/antiox11020366)
Supplement: Supplementary file 1 [file antioxidants-11-00366-s001.zip › antioxidants-1545932-supplementary.pdf]

ONLINE SUPPLEMENTAL MATERIALS

Prenatal fine particulate matter, maternal micronutrient antioxidant intake, and early childhood repeated wheeze: Effect modification by race/ethnicity and sex

Yueh-Hsiu Mathilda Chiu, ScD [1,2,3]; Kecia N. Carroll, MD, MPH [1,2,3]; Brent A. Coull, PhD [4]; Srimathi Kannan, PhD [5]; Ander Wilson, PhD [6]; Rosalind J. Wright, MD, MPH [1,2,3]

Author Affiliations:

[1] Department of Environmental Medicine and Public Health, Icahn School of Medicine at Mount Sinai, New York, NY, USA

[2] Kravis Children's Hospital, Department of Pediatrics, Icahn School of Medicine at Mount Sinai, New York, NY, USA

[3] The Institute for Exposomic Research, Icahn School of Medicine at Mount Sinai, New York, NY, USA

[4] Department of Biostatistics, Harvard TH Chan School of Public Health, Harvard University, Boston, MA

[5] Division of Metabolism, Endocrinology, and Diabetes, Department of Internal Medicine, University of Michigan, Ann Arbor, MI, USA

[6] Department of Statistics, Colorado State University, Fort Collins, CO, USA

Supplement to "2.1 Maternal Antioxidant Intake" section under MATERIALS AND METHODS:
Detailed Descriptions on the Selection of the Seven Antioxidant Micronutrients –
To derive a composite index of antioxidant intake, we considered seven micronutrients including β-carotene, magnesium, selenium, zinc, and vitamins A, C, and E. These seven micronutrients are well-established antioxidant compounds that meet the evidence-based criteria set forth by the Institute of Medicine (IOM) for dietary antioxidants which have been suggested to prevent damage caused by free radicals by neutralizing them (IOM 1998, 2000). More specifically, the following criteria support classification of the selected micronutrients as antioxidants:

(1) Vitamin C, Vitamin E, and Vitamin A – an established and evidence-based direct role as a dietary antioxidant, as defined by the Institute of Medicine which states that a dietary antioxidant is a substance in foods that significantly decreases the adverse effects of reactive species, such as reactive oxygen and nitrogen species, on normal physiological function in humans;
(2) Selenium, Magnesium and Zinc – required for the synthesis of an antioxidant or display indirect antioxidant activity as constituents of enzymes including superoxide dismutase;
(3) β-carotene – displays *in vitro* antioxidant activity.

Furthermore, our group has previously validated FFQ estimates of antioxidant intake for these micronutrients, demonstrating reasonable correlations between FFQ reports and 24-hour dietary recalls in PRISM participants (Brunst et al., 2016).

Supplement to "2.5 Statistical Analysis" section under MATERIALS AND METHODS:
Detailed Descriptions on the Implementation of Bayesian Distributed Lag Interaction Models (BDLIMs) –
We estimated antioxidant-specific (high vs. low intake based on media split of the composite antioxidant index (AI) score) and sex-specific time-varying associations between weekly $PM_{2.5}$ exposure and repeated wheeze using Bayesian distributed lag interaction models (BDLIMs) as previously described by our group (Wilson et al., 2017). This approach assumes that $PM_{2.5}$ effects in any given exposure timepoint is linear but allows effects to vary nonlinearly across exposure timepoints. We first fit models assuming a common distributed lag effect for all subjects, and then fit distributed lag interaction models to examine differences in both the magnitude and timing of effects by maternal antioxidant intake (high vs. low based on median split of AI score), as well as by child sex. Taking effect modification by antioxidant intake status as an example, the logistic BDLIM for child i ($i = 1, \dots, n$) whose mother's antioxidant intake status j ($j = 0$ for low intake and $j = 1$ for high intake) is $\mathrm{Logit}[E(Y_i)] = a_j + \beta_j \sum_{t=1}^{T} w_{jt} X_{it} + Z_i' \gamma$, where a_j is a fixed antioxidant-specific intercept, β_j is the regression coefficient characterizing the antioxidant-specific association between weighted $PM_{2.5}$ exposure and children's repeated wheeze status, $\sum_{t=1}^{T} w_{jt} X_{it}$ is the weighted exposure, with T denoting the number of timepoints, and $Z_i' \gamma$ is the covariate regression term. The w_{jt}, for $t = 1, \dots, T$, (weights) identify critical windows of susceptibility while β_j is the unweighted effect estimates (magnitudes). When weights are constant across time, this is equivalent to using mean exposure. However, when the weight varies by the time, the model assigns greater relative weight to more relevant periods. Time periods with weights substantially different from zero identify critical windows. The model uses a smooth orthonormal basis based on the joint distribution of the time-resolved exposure data to smooth the w_{jt} terms. Four patterns of effect modifications were examined by allowing the weights w_{jt} (critical windows) and β_j (effect magnitudes) to be specific for each level of the tested categorical effect modifier: 1) different critical windows and different

effect magnitudes; 2) different critical windows but same effect magnitude; 3) same critical window but different effect magnitudes; and 4) same critical window and same effect magnitude (i.e., no modification). We then further examined joint effect modification by both maternal antioxidant intake and child sex, where j in logistic BDLIM represents four possible combinations of antioxidant intake and child sex ($j = 0$ for low antioxidant/girls, $j = 1$ for low antioxidant/boys, $j = 2$ for high antioxidant/girls, and $j = 3$ for high antioxidant/boys). Posterior model probability and deviance information criterion (DIC) were used to determine the model that best fit the data.

Table S1: Comparison between participants enrolled in the PRISM Study and those included in the current analysis.

Comparison was done between those enrolled and those included in the analysis (i.e., who have complete data on prenatal daily ambient $PM_{2.5}$ exposure, daily temperature levels, dietary intake, and children's repeated wheeze status). Overall, most of the basic characteristics (child sex, maternal age, maternal education, and maternal smoking status) were similar between those enrolled and those included in the analysis, except that the composition of maternal race/ethnicity was different.

Figure S1: Associations between maternal antioxidant intake status (high vs. low) and children's repeated wheeze.

Results from multivariable-adjusted logistic regressions examining associations between maternal antioxidant intake, categorized as high vs. low based on median split of the composite antioxidant index (AI) score, and children's repeated wheeze in the sample overall and stratified by race/ethnicity. The association patterns were similar to the models using continuous AI score as shown in Figure 2 in the main manuscript, but statistical significance was attenuated.

Figure S2: Time-varying odds ratio (95% CI) of repeated wheeze corresponding to per $\mu g/m^3$ increase in prenatal weekly $PM_{2.5}$ levels.

BDLIM did not identify statistically significant $PM_{2.5}$ exposure windows for repeated wheeze in the PRISM sample overall.

Figure S3. Antioxidant-specific time-varying odds ratios (95% CIs) of repeated wheeze corresponding to per $\mu g/m^3$ increase in prenatal weekly $PM_{2.5}$ levels among children born to Black mothers.

BDLIM suggested that $PM_{2.5}$ exposure was associated with repeated wheeze in children born to Black mothers with low antioxidant intake, but models did not identify a statistically significant sensitive window (i.e., all estimated pointwise 95% CI represented by the shaded area included 1).

Figure S4. Sex-specific time-varying odds ratios (95% CIs) of repeated wheeze corresponding to per $\mu g/m^3$ increase in prenatal weekly $PM_{2.5}$ levels.

BDLIM did not identify a significant sensitive exposure window when examining sex-specific associations between prenatal $PM_{2.5}$ and repeated wheeze.

Figures S5. Antioxidant- and sex-specific time-varying odds ratios (95% CIs) of repeated wheeze corresponding to per μg/m^3 increase in prenatal weekly $PM_{2.5}$ levels: stratified by maternal race/ethnicity.

BDLIMs did not identify statistically significant antioxidant- and sex-specific exposure windows for repeated wheeze among Hispanic and White/Other subgroups. The estimated pointwise 95% CI (shaded area) included 1 for all timepoints across pregnancy in these two racial/ethnic subgroups. In addition, the confidence intervals are relatively wide and less stable in these subgroups as compared to Black subgroup, likely due to smaller sample sizes.

Figure S6. Odds ratios (95% CIs) of offspring's repeated wheeze corresponding to $PM_{2.5}$ exposure averaged over the entire gestational period in Black mothers, stratified by antioxidant intake and child sex.

When using $PM_{2.5}$ levels averaged over the entire pregnancy period in the multivariable-adjusted logistic regressions, we did not observe statistically significant associations between prenatal $PM_{2.5}$ and repeated wheeze.

Figures S7 & S8. Antioxidant- and sex-specific time-varying odds ratios (95% CIs) of repeated wheeze corresponding to per μg/m^3 increase in prenatal weekly $PM_{2.5}$ levels among children born to Black mothers – sensitivity analyses adjusting for additional covariates.

Sensitivity analyses were conducted by performing BDLIMs in Black group, which additionally adjusted for prenatal maternal smoking, secondhand smoke exposure, postnatal PM2.5 level averaged over the first year of child life (Figure S8), as well as further adjusting for study site, season of birth, and birthweight for gestational age z-score (Figure S9), on top of the originally included covariates maternal age at delivery, education, asthma history, and prenatal averaged temperature (main manuscript, Figure 3). The results of these sensitivity analyses were materially unchanged.

Supplemental Table S1. Participant Characteristics: PRISM Study

	Included in Analysis (N=530)		Enrolled Participants (N=923)		p-value [b]
**Maternal age at delivery**					
Age in years (median, IQR [a])	30.2	(25.3–34.2)	29.4	(24.7–33.6)	0.12
**Child sex (n, %)**					
Female	254	47.9	441	47.8	0.96
Male	276	52.1	482	52.2	
**Maternal education (n, %)**					
>12 years	342	64.5	531	57.5	0.18
≤12 years	188	35.5	341	36.9	
Missing	-	-	51	5.5	
**Maternal race/ethnicity (n, %)**					
Black/Hispanic-Black	205	38.7	375	40.6	0.02
Non-Black Hispanic	175	33.0	319	34.6	
Non-Hispanic White	120	22.6	145	15.7	
Other	30	5.7	41	4.4	
Unknown/Missing	-	-	43	4.7	
**Maternal history of asthma**					
No	372	70.2	607	65.8	0.87
Yes	158	29.8	263	28.5	
Missing	-	-	53	5.7	

[a] IQR = interquartile range (25th percentile – 75th percentile).

[b] p-values of the tests comparing the available (non-missing) data between those enrolled vs. included in analysis. Wilcoxon ranked sum test for continuous variables, and χ^2 test for categorical variables.

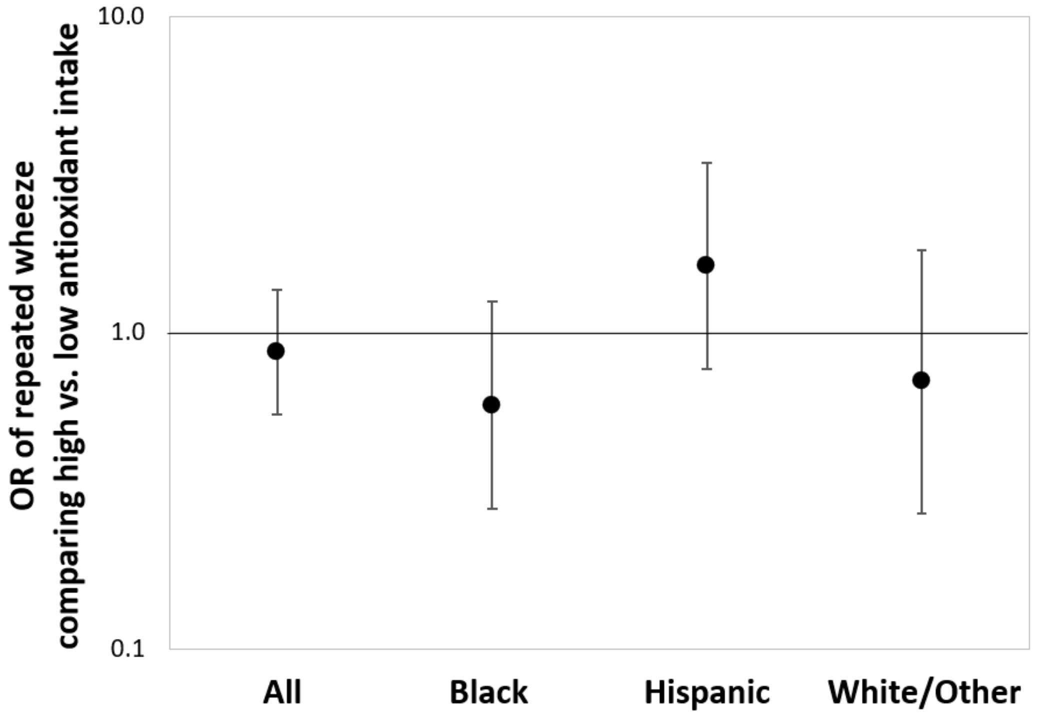

Supplemental Figure S1. Associations between maternal antioxidant intake status (high vs. low) and children's repeated wheeze

Results from multivariable-adjusted logistic regressions examining associations between maternal antioxidant intake status (high vs. low based on median split of the composite AI score) and children's repeated wheeze in the sample overall and stratified by race/ethnicity (Black: Black/Hispanic-Black; Hispanic: non-Blank Hispanic; White/Other: non-Hispanic White/other race). Solid dot denotes odds ratio (OR) of repeated wheeze comparing high vs. low antioxidant intake, and the error bars denote 95% confidence interval (95% CI). The models were adjusted for child sex, maternal age at delivery, maternal education status, maternal history of asthma (and race/ethnicity in the overall model).

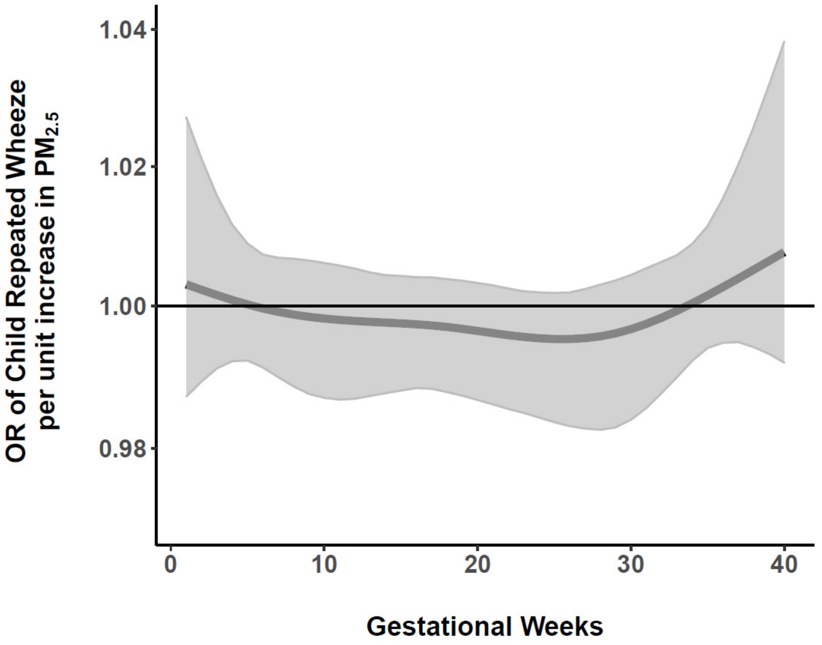

Supplemental Figure S2. Time-varying odds ratio (95% CI) of repeated wheeze per µg/m^3 increase in prenatal weekly averaged $PM_{2.5}$ levels.

Time-varying associations between prenatal weekly $PM_{2.5}$ exposure and children's repeated wheeze were estimated by a BDLIM, adjusting for maternal age at delivery, education, race/ethnicity, history of asthma, prenatal averaged temperature, and child sex. The x-axis demarcates gestational age in weeks. The y-axis represents the odds ratio (OR) of repeated wheeze per 1 µg/m^3 increase in $PM_{2.5}$ exposure. The solid line represents the predicted OR, and the gray area indicates the 95% confidence interval (CI).

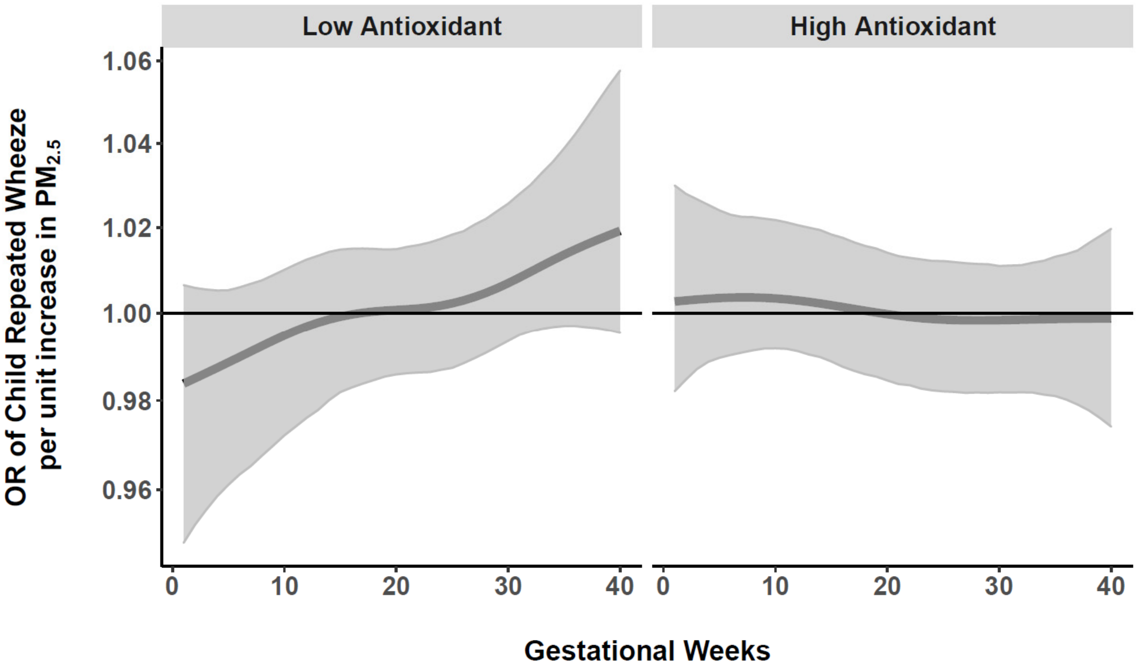

Supplemental Figure S3. Antioxidant-specific time-varying odds ratios (95% CIs) of repeated wheeze per µg/m^3 increase in prenatal weekly averaged $PM_{2.5}$ levels among children born to Black mothers.

Antioxidant-specific time-varying associations between prenatal weekly $PM_{2.5}$ exposure and children's repeated wheeze were estimated by a BDLIM among Black (black and Hispanic-black), adjusting for maternal age at delivery, education, history of asthma, prenatal averaged temperature, and child sex. The x-axis demarcates gestational age in weeks. The y-axis represents the odds ratio (OR) of repeated wheeze per 1 µg/m^3 increase in prenatal $PM_{2.5}$ exposure. The solid line represents the predicted OR, and the gray area indicates the 95% confidence interval (CI).

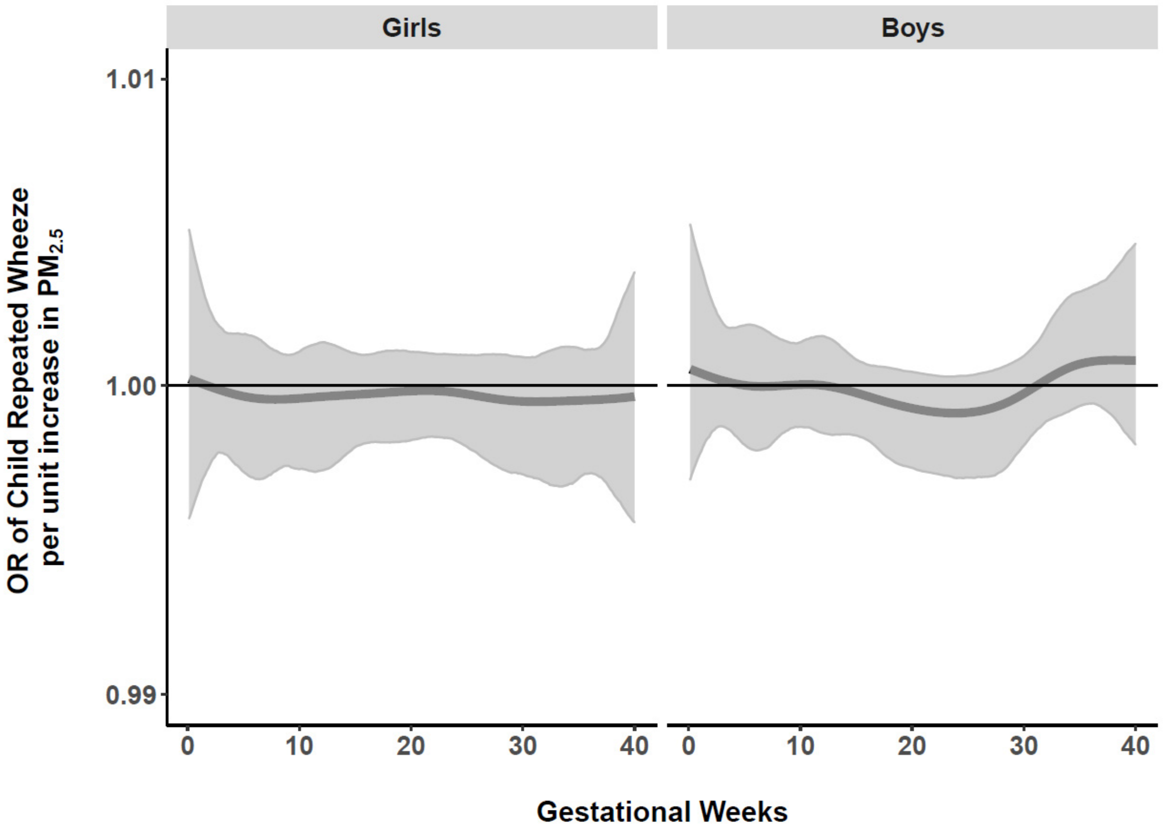

Supplemental Figure S4. Sex-specific time-varying odds ratios (95% CIs) of repeated wheeze per µg/m^3 increase in prenatal weekly averaged $PM_{2.5}$ levels.

Sex-specific time-varying associations between prenatal weekly $PM_{2.5}$ exposure and children's repeated wheeze were estimated by a BDLIM in the overall sample, adjusting for maternal age at delivery, education, history of asthma, prenatal averaged temperature, and maternal race/ethnicity. The x-axis demarcates gestational age in weeks. The y-axis represents the odds ratio (OR) of repeated wheeze per 1 µg/m^3 increase in prenatal $PM_{2.5}$ exposure. The solid line represents the predicted OR, and the gray area indicates the 95% confidence interval (CI).

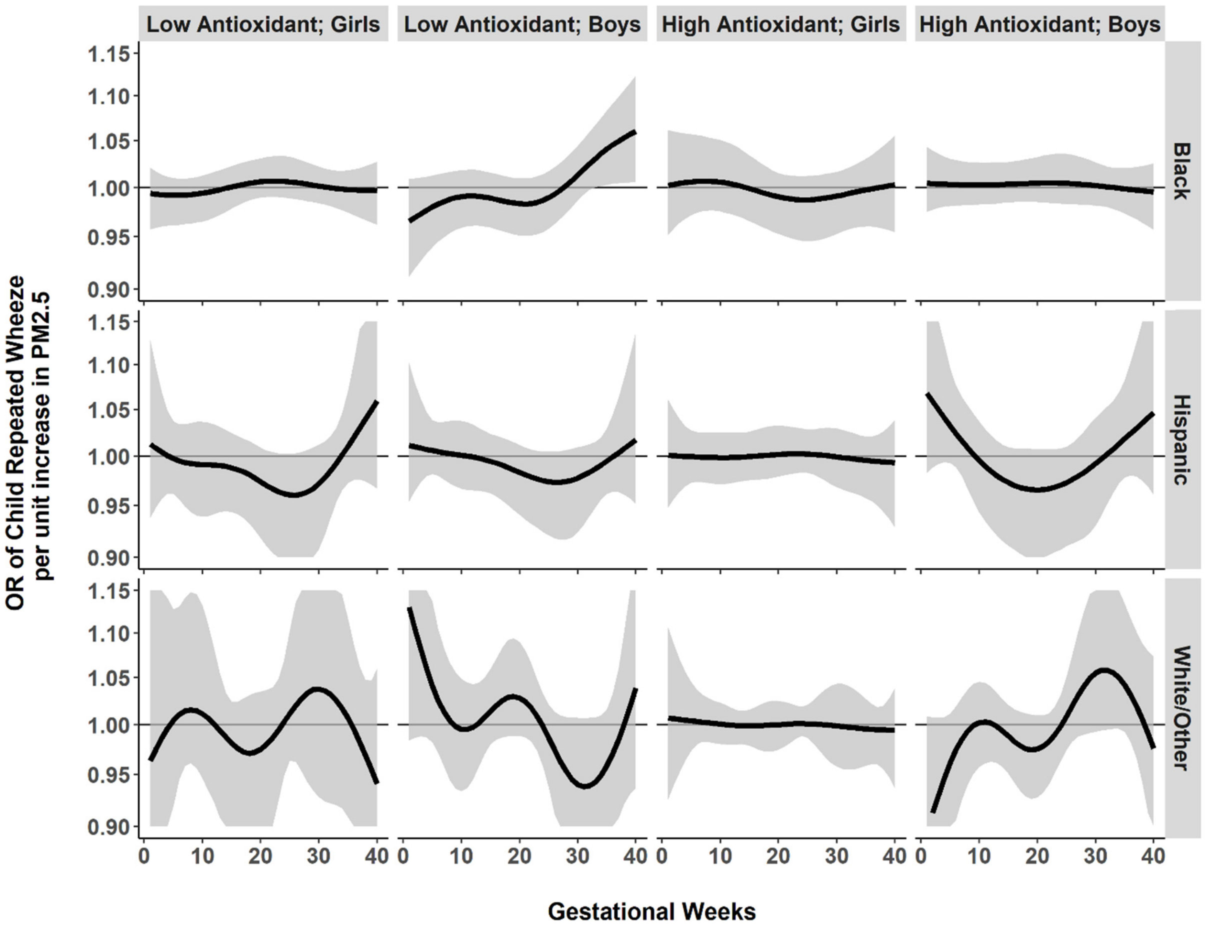

Supplemental Figure S5. Antioxidant- and sex-specific time-varying odds ratios (95% CIs) of repeated wheeze per µg/m^3 increase in prenatal weekly averaged $PM_{2.5}$ levels: stratified by maternal race/ethnicity.

Antioxidant-specific time-varying associations between prenatal weekly $PM_{2.5}$ exposure and children's repeated wheeze were estimated by a BDLIM for each racial/ethnic subgroups. The top panel (for Black group) is the same as Figure 3 in the main manuscript, while the 2nd and 3rd panels are the BDLIM results among Hispanic and White/Other subgroups, respectively. Models were adjusting for maternal age at delivery, education, history of asthma, and prenatal averaged temperature. The x-axis demarcates gestational age in weeks. The y-axis represents the odds ratio (OR) of repeated wheeze per 1 µg/m^3 increase in prenatal $PM_{2.5}$ exposure. The solid line represents the predicted OR, and the gray area indicates the 95% confidence interval (CI). Note that the confidence intervals are relatively wide and less stable for Hispanic and White/Other models compared to the model conducted in Blacks, likely due to smaller sample sizes.

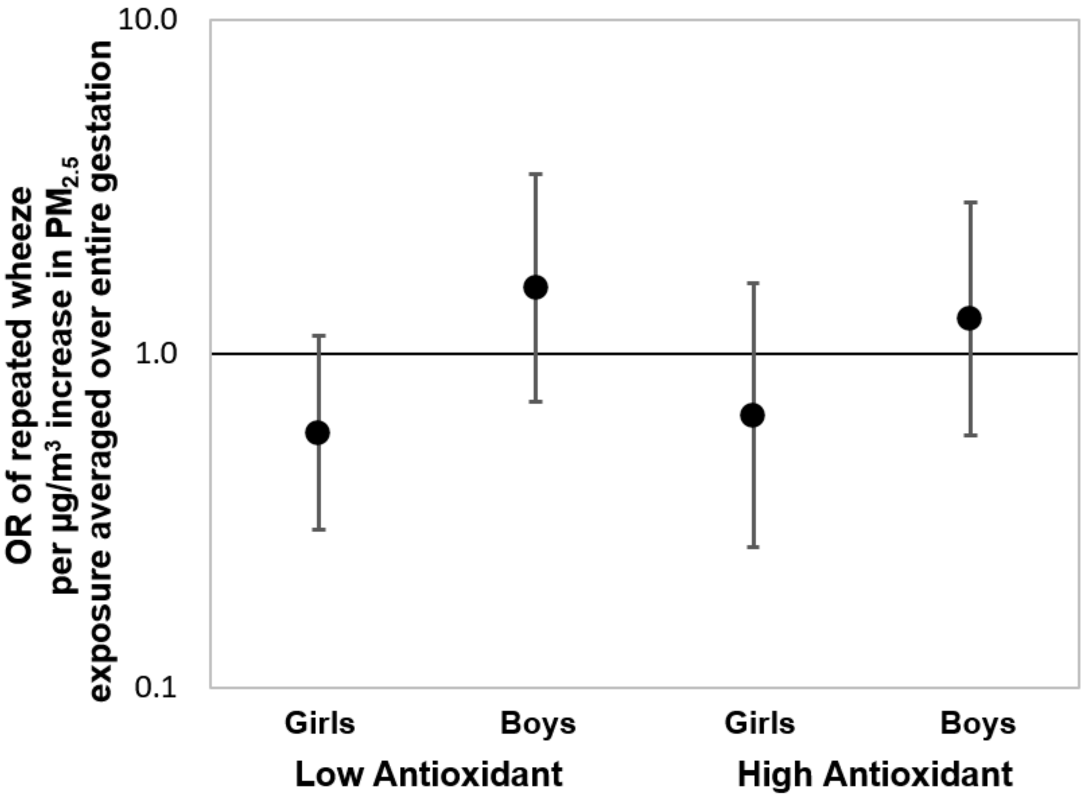

Supplemental Figure S6. Odds ratios (95% CIs) of offspring's repeated wheeze corresponding to $PM_{2.5}$ exposure averaged over the entire gestational period in Black mothers, stratified by antioxidant intake and child sex.

Results from multivariable-adjusted logistic regressions examining the associations between prenatal $PM_{2.5}$ exposure level averaged over the entire gestation period and offspring's repeated wheeze among Black (Black and Hispanic-Black) mothers, stratified by antioxidant intake status (high vs. low intake categorized by median split) and child sex. The models were adjusted for maternal age at delivery, maternal education, maternal history of asthma, and prenatal averaged temperature.

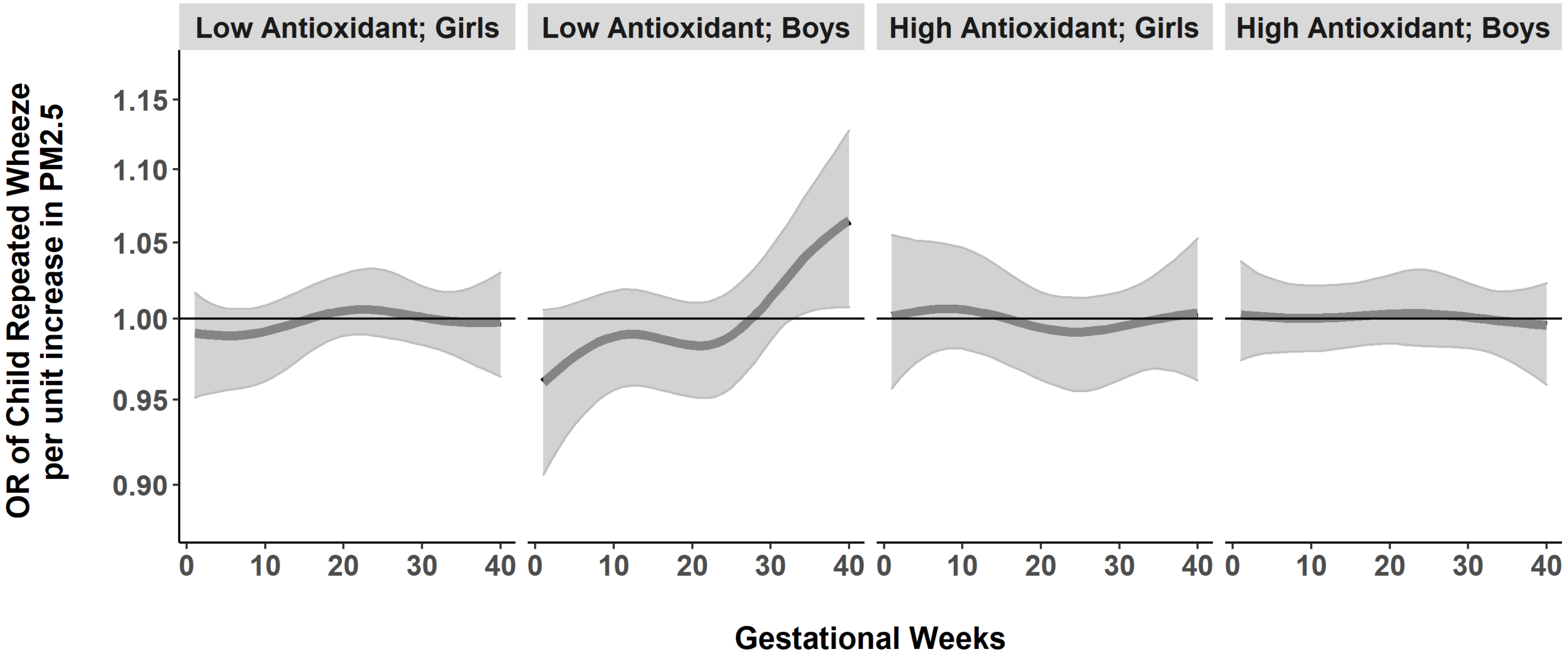

Figure S7. Antioxidant- and sex-specific time-varying odds ratios (95% CIs) of child's repeated wheeze per μg/m^3 increase in prenatal weekly averaged $PM_{2.5}$ levels across gestation in Black mothers: Sensitivity Analysis (1).

Antioxidant- and sex-specific time-varying associations between prenatal weekly $PM_{2.5}$ exposure and children's repeated wheeze were estimated by a BDLIM among Black mothers, adjusting for maternal age at delivery, education, asthma history, prenatal averaged temperature, prenatal maternal smoking, secondhand smoke exposure, and postnatal $PM_{2.5}$ level averaged over the first year of child life. The x-axis demarcates gestational age in weeks. The y-axis represents the odds ratio (OR) of repeated wheeze per 1 μg/m^3 increase in prenatal $PM_{2.5}$ exposure. The solid line represents the predicted effect estimate, and the gray area indicates the 95% confidence interval (CI). A significant exposure window is identified for the time periods where the estimated pointwise 95% CI (shaded area) does not include 1. Results indicated a significant exposure window at 33-40 weeks gestation in boys born to Black mothers with low antioxidant intake.

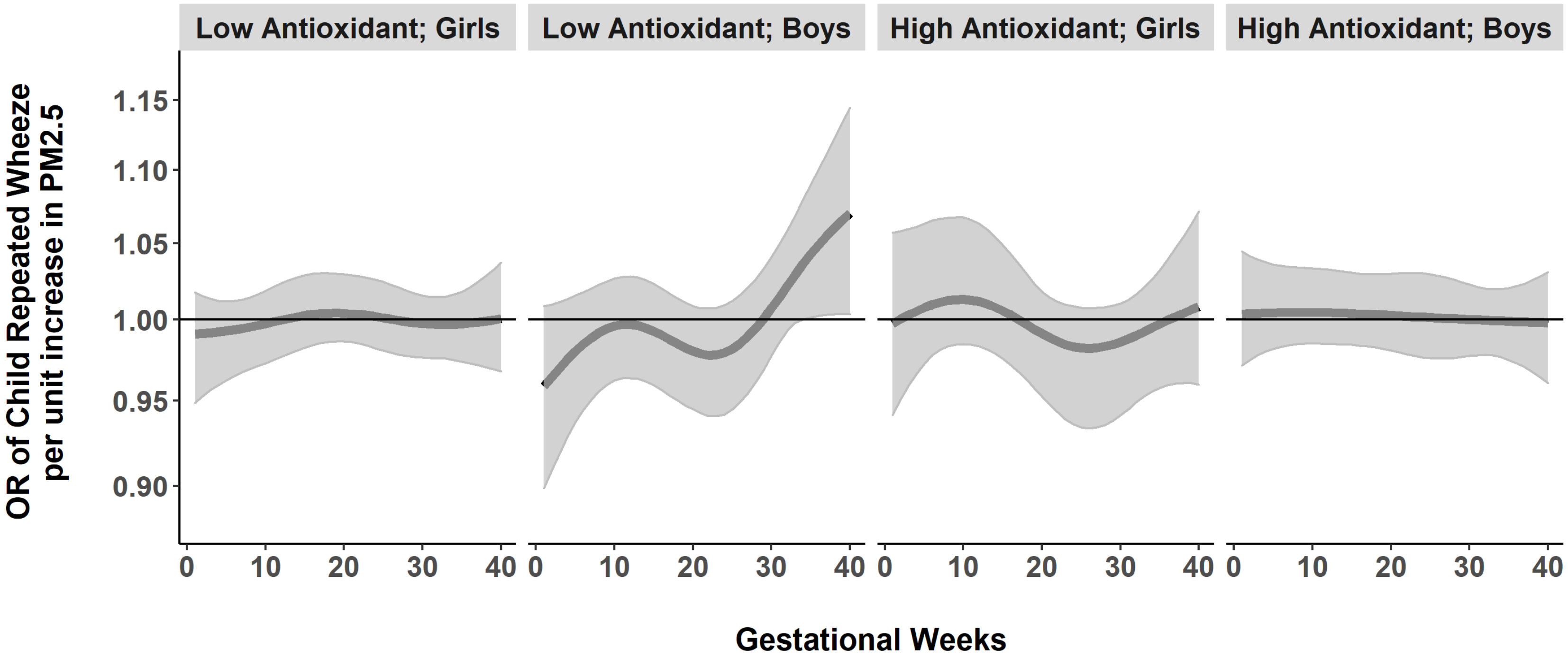

Figure S8. Antioxidant- and sex-specific time-varying odds ratios (95% CIs) of child's repeated wheeze per μg/m^3 increase in prenatal weekly averaged $PM_{2.5}$ levels across gestation in Black mothers: Sensitivity Analysis (2).

Antioxidant- and sex-specific time-varying associations between prenatal weekly $PM_{2.5}$ exposure and children's repeated wheeze were estimated by a BDLIM among Black mothers, adjusting for maternal age at delivery, education, asthma history, prenatal averaged temperature, prenatal maternal smoking, secondhand smoke exposure, postnatal $PM_{2.5}$ level averaged over the first year of child life, study site, season of birth, and birthweight for gestational age z-score. The x-axis demarcates gestational age in weeks. The y-axis represents the odds ratio (OR) of repeated wheeze per 1 μg/m^3 increase in prenatal $PM_{2.5}$ exposure. The solid line represents the predicted effect estimate, and the gray area indicates the 95% confidence interval (CI). A significant exposure window is identified for the time periods where the estimated pointwise 95% CI (shaded area) does not include 1. Results indicated a significant exposure window at 33-40 weeks gestation in boys born to Black mothers with low antioxidant intake.

REFERENCES:

Brunst, K.J., Kannan, S., Ni, Y.M., Gennings, C., Ganguri, H.B., Wright, R.J., 2016. Validation of a Food Frequency Questionnaire for Estimating Micronutrient Intakes in an Urban US Sample of Multi-Ethnic Pregnant Women. Matern Child Health J. 20, 250-260.

IOM, 1998. Institute of Medicine (IOM) Standing Committee on the Scientific Evaluation of Dietary Reference Intakes. Dietary Reference Intakes: Proposed Definition and Plan for Review of Dietary Antioxidants and Related Compounds. Washington (DC): National Academies Press (US).

IOM, 2000. Institute of Medicine (IOM) Panel on Dietary Antioxidants Related Compounds. Dietary Reference Intakes for Vitamin C, Vitamin E, Selenium, and Carotenoids. Washington (DC): National Academies Press (US).

Wilson, A., Chiu, Y.H.M., Hsu, H.L., Wright, R.O., Wright, R.J., Coull, B.A., 2017. Bayesian distributed lag interaction models to identify perinatal windows of vulnerability in children's health. Biostatistics. 18, 537-552.
